# Spontaneous Coronary Artery Dissection in Patients With Fibromuscular Dysplasia

**DOI:** 10.7759/cureus.13696

**Published:** 2021-03-04

**Authors:** Mahboob Alam, Ozo Akah, Tuba Khan, Raza Zarrar

**Affiliations:** 1 Cardiology, Baylor College of Medicine, Houston, USA; 2 Internal Medicine, Carnegie Mellon University, Houston, USA; 3 Medicine and Surgery, Ziauddin Medical College, Karachi, PAK; 4 Medicine, Ghurki Hospital, Lahore, PAK

**Keywords:** scad management, fmd and scad

## Abstract

Clinicians must be mindful of angiographic appearances in patients with spontaneous coronary artery dissection (SCAD) in the setting of fibromuscular dysplasia (FMD) for the timely management of these high-risk patients. The objective is to highlight the clinical diagnostic and treatment modalities in rare case presentations of patients presenting with concurrent SCAD and FMD presentation. A qualitative review of scholarly materials. Twenty-seven patients who presented with a combination of SCAD and FMD from January 1, 2009, to August 2019 were identified. Various demographics such as age, gender, FMD location, acute-phase treatment (i.e., percutaneous coronary intervention (PCI) vs. coronary artery bypass grafting (CABG) vs. conservative), treatment outcomes, and then grouped into two tables. The mean age >46 years and standard deviation (SD) were used to calculate the normal distribution and percentile used to calculate others for treatment. SCAD and FMD cases were collected from three search engines ranging between 2009 and 2019. 22% of the patients had coronary artery disease (CAD). Additionally, 44.4% representing 12 patients with ST-segment elevation acute myocardial infarction (STEMI), four patients 14.8% presented with a non-ST-segment elevation myocardial infarction (NSTEMI), and nine patients 33.33% offered with unstable angina. Besides, 13 patients were diagnosed with optical coherence tomography (OCT), while intravascular ultrasound (IVUS) diagnosed six patients. SCAD is still very rare compared to other causes of myocardial infarction. Data has shown that up to 25% of acute coronary syndrome (ACS) cases of women between 40 and 65 years are SCAD.

## Introduction and background

Spontaneous coronary artery dissection (SCAD) is a non-traumatic, non-iatrogenic, and non-atherosclerotic separation of the coronary arterial wall resulting in the formation of a false lumen within the vessel wall with subsequent accumulation of blood. This process leads to compression of the true lumen, causing symptoms like those of myocardial infarction [1]. Fibromuscular dysplasia (FMD) is an idiopathic, segmental, non-inflammatory, and non-atherosclerotic disease of the arterial wall that displays as a characteristic ‘string of beads’ on imaging equipment. FMD is mainly present in women between 30 and 50 years of age [2]. The majority of patients with SCAD have concomitant FMD, and the strong association between them is unquestionably convincing for a causative relationship. The diagnosis and management of SCAD may be insufficient, since an angiographic study cannot visualize the arterial walls. Therefore, intracoronary imaging modalities like optical coherence tomography (OCT) or intravascular ultrasound (IVUS) are often used to diagnose the presence of SCAD [3]. Given the lack of large-scale studies and the rare occurrence of FMD and SCAD in the general population, we performed a systematic and scientific review of existing literature to better define disease presentation, management, and clinical outcomes [4].

Methods

The study used Google Scholar, PubMed, and Embase to locate qualifying case studies. We identified 27 patients who presented with both SCAD and FMD. The findings were based on a search performed for cases published between January 1, 2009 and January 1, 2019. Patient information was extracted from the cases evaluated in this study. If either of the following were the case, the study was not included:

Data available for SCAD, but not FMD.

Data available for both SCAD and FMD, but with insufficient patient information to evaluate SCAD.

The following data were extracted from the studies, where possible: patient gender and age, the presence of diabetes (DM), hyperlipidemia (HDL), hypertension (HTN), coronary artery disease (CAD), SCAD, FMD locations, electrocardiogram (EKG), levels of troponin, follow-up after discharge, angiographic findings, treatment, adverse events, length of hospital, and treatment outcomes. This was assembled into two tables, which serve as a descriptive summary of the study features and clinical outcomes, including means and scopes with standard deviations offered for definite variables. The study was conducted using SPSS version 23.0 software (IBM Corp., Armonk, NY).

## Review

Results

The median age of patients was 46.4±-8.5 years (see Table 1). Of all the patients included in this study, 22% had CAD, and the following risk factors were reported: HTN 14.8%, DM 3.7%, and HLD 14.8%. The majority of the patients were female, representing 26 out of 27 study participants. Twelve patients (44.4%) presented with segment elevation acute myocardial infarction (STEMI), four patients (14.8%) presented with NSTEMI, and nine patients (33.33%) presented with unstable angina. LCX (9/27) and LAD (21/27) were the SCAD angiographic locations, with subsequent descending artery 4/27, vertebral artery, and 4/27 left main artery (Table 2). Optical coherence tomography (OCT) and intravascular ultrasound (IVUS) aided diagnosis confirmation and guided the management of all the cases. 

**Table 1:** Patient characteristics, comorbidities, and FMD locations of adult patients (N = 27) with SCAD. SD of S: standard deviation of sample; N: total number of participants; CAD: coronary artery disease; HTN: hypertension; HLD: hyperlipidemia; VA: vertebral artery; DM: diabetes mellitus; ICA: internal carotid artery; FMD: fibromuscular dysplasia; SCAD: spontaneous coronary artery dissection. *Nonspecific carotid artery, including one ICA.

		N (27)	Mean	SD of S
Characteristics	Age (years)		46.96	8.702
	Female		0.96	0.192
	Male		0.04	0.192
Comorbidities	CAD	6 (22.22%)		
	HLD	4 (14.81%)		
	HTN	4 (14.81%)		
	DM	1(3.70%)		
FMD location	Renal artery	18 (66.67%)		
	Iliac artery	12 (44.44%)		
	VA	9 (33.33%)		
	Carotid*	9 (33.33%)		
	Femoral	1(3.70%)		

**Table 2:** Arterial dissection location. ACS: acute coronary syndrome; *Main includes ramus artery; PDA includes interventricular artery. IVUS: intravascular ultrasound; OCT: optical coherence tomography; STEMI: segment elevation acute myocardial infarction; NSTEMI: non-ST-segment elevation myocardial infarction.

		N (27)
Location of artery	Left anterior descending (LAD)	21 (77.78%)
	Right coronary artery (RCA)	5 (18.52%)
	Left circumflex artery (LCX)	9 (33.33%)
	Left main coronary artery*	6 (22.22%)
Intra-coronary Imaging	OCT	13 (48.15%)
	IVUS	6 (22.22%)
ACS location	Unstable angina	9 (33.33%)
	STEMI	12 (44.44%)
	NSTEMI	4 (14.81%)

Thirteen additional patients were diagnosed with OCT, while six were diagnosed with IVUS. Among the 27 patients, 17 initially managed conservatively, and eight were treated with percutaneous coronary intervention (PCI) based on the severity of initial symptoms. Among patients that received PCI, four were treated primarily with drug-eluting stents, with two of the patients who already had drug-eluting stents receiving additional drug-eluting stents before discharge (Table 3). Further, one patient was managed with both PCI and conservative treatment, none of the patients underwent coronary artery bypass grafting, and one patient died during the administration of an intra-aortic balloon pump. Coronary artery grafting was not indicated for any of the patients. After discharge, two (7.41%) of the patients experienced four more recurrences that were addressed with conventional treatment. Most patients exhibited artery healing without any recurrence.

**Table 3:** SCAD-FMD treatment outcome in stable and unstable patients. PCI: percutaneous coronary intervention; CABG: coronary artery bypass grafting; SCAD: spontaneous coronary artery dissection; FMD: fibromuscular dysplasia.

		N (27)
Management types (stable patients)	Conservative	17 (69.96%)
	No. of patients with multiple recurrent events	2/17 (11.76%)
	Total no. of recurrent events	8
	Number of recurrences managed conservatively	4
Management types (unstable patients)	PCI	8 (29.63%)
	PCI with drug stent	4/8 (50.00%)
	Pt with ≥2 stents prior to discharge	2/8 (25.00%)
	Death prior to discharge	1/27(3.70%)
	CABG	0/27

Discussion

In our analysis of SCAD and FMD patients with a median age of 46.4±8.5 years, 96% of patients were female with a mean age of 46.96 and SD of 8.7 years (Table 1), which agrees with existing literature. This suggests that clinicians should consider the possibility of SCAD in middle-aged females who present with acute coronary syndrome (ACS), despite the absence of traditional risk factors for CAD. SCAD is an uncommon genetic vascular disease affecting the arteries of the body and occurs when areas of artery narrow alternating with areas of dilation, non-atherosclerotic etiology [5]: the typical risk factors for heart disease, including hypertension, hyperlipidemia, diabetes mellitus, smoking, obesity, and a family history of premature CAD, may not be observed [6]. The results of our analysis show that only 3.7% of patients had DM, while 14.8% of patients had hypertension or hyperlipidemia (Table 1). Multivessel dissections with the involvement of left anterior descending (77.78%) and right coronary (8.52%) arteries were most commonly observed (Table 2). 

According to our findings, the predominant arteries involved were renal (66.67%), iliac (44.44%), carotid (37.04%), and vertebral (33.33%) (Table 1). Eighty-six percent of cases of SCAD patients had concomitant FMD, which might suggest shared pathophysiology. The pathophysiology of FMD-causing SCAD remains unknown; however, it is likely due to an underlying genetic abnormality, such as fibrosis of the vasa vasorum leading to coronary vessel wall ischemia and myofibroblast proliferation, resulting in dissection [7,8]. The hallmark of this type of FMD is the ‘string of beads’ appearance of the arteries (see images in [5]).

Since coronary angiography is the gold-standard imaging modality for patients presenting with ACS, SCAD patients must undergo the same procedure. Unfortunately, angiography has several limitations, including the inability to view the layers and structures of the coronary arterial wall [6]. Therefore, advanced intracoronary imaging tools such as OCT, IVUS, or multidetector CT can be used to diagnose, measure intramural hematoma flow, and track dissection over time [1].

An association between SCAD and FMD has been speculated based upon numerous published case reports [4,9-25]; however, there is a lack of guidelines for diagnosis and treatment in the acute presentation of SCAD. Hence, most recommendations are based purely on expert opinion. Some clinicians (69.96%) preferred a conservative approach in hemodynamically stable patients, with recurrence of SCAD in only 11.76%. In contrast, hemodynamically unstable patients were treated invasively with PCI (29.63%) (Table 1). Conservative therapy involves treatment with aspirin and beta-blockers to modify shear forces across delicate arterial segments. The role of antiplatelet agents and anticoagulants in SCAD has not been well studied, but dual antiplatelet therapy may also be administered for one year, followed by lifelong aspirin for secondary prevention. Conservative treatment is preferred since the false lumen containing the intramural hematoma will be reabsorbed over time [1,3]. PCI or CABG may be performed, depending on the clinical presentation and the degree of compromise to coronary flow in an angiographic study [26]. 

Limitations of the study

All cases used for the study were retrospective. This meant participants were not enrolled specifically for the purposes of the study and testing and follow-up were conducted based on individual circumstances, which makes them nonuniform. Additionally, this may not reflect SCAD in the general population as more serious patients might have been subjected to testing and follow-up. Follow-up was over a limited period of time, hence no deduction on long-term sequelae could be made. Follow-up was inconsistent in some cases, so it might be possible that some patients had a recurrence and were not reported. The study had a relatively limited number of cases which reduces its power. Additionally, the overall mortality in women is relatively low, however, such data is not known for male patients.

## Conclusions

Our analysis included 27 patients with SCAD with FMD spanning 10 years. SCAD is a relatively rare cause of ACS. Recently, there has been an increase in the detection of SCAD. SCAD accounts for up to 25% of ACS cases in women between the ages of 40 and 65. Outcomes for patients with SCAD were mostly positive, except for one patient who died during intra-aortic balloon pump therapy. Additional research is improving our understanding of SCAD; however, there is a need for treatment consensus. Based on our analysis, it is essential to differentiate SCAD from other causes of ACS upon initial presentation with the help of history and imaging. Considering that SCAD commonly occurs in association with arteriopathies and FMD, an early systemic vascular workup should be performed on such patients. Early diagnosis may save patients from unnecessary and destructive treatment. Further research is warranted on the mean age of more than 50 years.
